# Ion-Imprinted Polypropylene Fibers Fabricated by the Plasma-Mediated Grafting Strategy for Efficient and Selective Adsorption of Cr(VI)

**DOI:** 10.3390/polym11091508

**Published:** 2019-09-16

**Authors:** Zhengwei Luo, Jiahuan Xu, Dongmei Zhu, Dan Wang, Jianjian Xu, Hui Jiang, Wenhua Geng, Wuji Wei, Zhouyang Lian

**Affiliations:** 1 College of Biotechnology and Pharmaceutical Engineering, Nanjing Tech University, 30# Puzhu South Road, Nanjing 211816, China; luozw1989@163.com (Z.L.); jianghuinjtech2018@163.com (H.J.); gengwenhua2018@163.com (W.G.); 2 School of Environmental Science and Engineering, Nanjing Tech University, 30# Puzhu South Road, Nanjing 211816, China; xu_jiahuan2018@163.com (J.X.); dongmei_zhunjtech@163.com (D.Z.); wangdannjtech@163.com (D.W.); xujianjian@163.com (J.X.)

**Keywords:** polypropylene, nonwoven fibers, plasma, imprinted polymer, chromium

## Abstract

To improve the adsorption selectivity towards hexavalent chromium anion (Cr(VI)), surface Cr(VI)-imprinted polypropylene (PP) fibers were fabricated by the plasma-mediated grafting strategy. Hence, a non-thermal Rradio frequency discharge plasma irradiation followed by a gaseous phase grafting was used to load acrylic acid (AA) onto PP fibers, which was afterwards amidated with triethylenetetramine and subjected to imprinting with a Cr(VI) template. The plasma irradiation conditions, i.e., gas species, output power, pressure, and time, were optimized and then the influence of grafting time, pressure, and temperature on the grafting degree of AA was investigated. Scanning electron microscopy and Fourier transform infrared spectroscopy were used for the characterization of pristine and modified fibers and to confirm the synthesis success. The hydrophilicity of modified fibers was greatly improved compared with pristine PP fibers. The adsorption thermodynamics and kinetics of Cr(VI) were investigated, as well as the elution efficiency and reusability. The prepared imprinted fibers showed superior adsorption selectivity to Cr(VI) compared with non-imprinted fibers. Finally, the stability of the imprinted fibers against the oxidation ability of Cr(VI) is discussed.

## 1. Introduction

Electroplating, metallurgy, and leather tanning industries produce a large amount of waste water containing hexavalent chromium anions (Cr(VI)), mainly in the form of oxyanions (e.g., HCrO_4_^−^). Cr(VI) has a strong oxidation character and mobility, as well as strong carcinogenic, teratogenic, and mutagenesis effects on humans [1,2]. Therefore, industrial wastewater containing a high concentration of Cr(VI) must be treated before being discharged. The usual methods include chemical reduction [3], membrane separation [4], the photocatalytic method [5], microbial removal [6], and adsorption. Among the chemical methods, the most commonly used are reduction and precipitation, but the cost is high and the sludge treatment is difficult, frequently causing secondary pollution. The recycling and utilization of Cr(VI) is considered to be the most environmentally friendly and economical approach [7]. Due to its advantages, such as high removal efficiency, low treatment cost, and reusability, the adsorption is one of the most efficient methods for the removal of Cr(VI) [8,9]. Although many adsorbents have been used for the adsorption of Cr(VI), there are still shortcomings to overcome, such as the adsorption capacity, adsorption rate, stability, and selectivity [10]. For instance, in the presence of competing ions, e.g., sulfate and phosphate, it is difficult to achieve a high adsorption capacity for Cr(VI) with the currently used adsorbents. Hence, there is a real demand for development of novel adsorbents.

Polypropylene (PP) melt-blown non-woven fabric has been widely used as an adsorbent due to its excellent chemical stability and good flexibility [11]. Grafting modification allows the polymerization of other monomers with polarity and functionality to the molecular chain of PP, which is the most effective way to improve the surface chemistry of PP. The plasma method requires mild process conditions, it is environmentally friendly, and it does not damage the internal structure of polymer materials. The principle of plasma grafting consists of using ionized high-energy particles to bombard the surface of the material to produce active sites (e.g., free radicals), triggering the grafting reaction of monomers on the surface. Grafting modification of PP fibers can greatly improve the hydrophilicity, functionality, and other physical and chemical properties [12]. Basarir et al. found that the electrochemical properties of PP films were improved as a result of activation with plasma followed by polymerization of the acrylic monomers on the surface [13]. Chen et al. grafted glycidyl methacrylate (GMA) on the surface of PP fibers, and the chelated fibers effectively adsorbed Cd(II) from water [14]. Tseng et al. used plasma to treat PP fibers on which GMA was grafted [15]. Then, the chelating fibers were modified with diethanolamine for Ag^+^ adsorption. Generally, gaseous plasma treatment and grafting of a monomer from the liquid phase are applied into two steps [16,17,18] or the plasma discharge treatment and grafting process occur simultaneously in monomer solution [19,20]. If the monomer gas is directly used for plasma pretreatment and grafting, the preparation steps can be simplified and the grafting efficiency can be improved [21].

Surface grafting modification of PP fibers for the adsorption of heavy metal ions can take advantage of the excellent properties of the polymer fibers, and high efficiency and selectivity of ion adsorption can be further achieved by using the surface ion-imprinted polymers. The essence of ion-imprinted polymers is to create specific adsorption sites on the surface, so as to improve the adsorption selectivity and capacity. The preparation steps generally include the preparation of polymeric adsorbent, adsorption of template metal ions, crosslinking, and elution of heavy metal templates [22]. Li et al. grafted the polyacrylic acid onto PP fiber surfaces followed by the reaction with polyethylene imine to prepare a Cu^2+^-imprinted polymer [23]. The resulted adsorbent exhibited good selectivity and regenerability. Zhang et al. prepared surface imprinted PP fibers induced by ^60^Co radiation to selectively adsorb uranyl carbonate from the seawater [24,25].

Plasma modification has been used to improve the adsorption performance of PP fibers for the removal of heavy metal cations. However, few studies have been conducted on the preparation of PP fiber-imprinted material by plasma grafting and its application for the adsorption of high valent oxyanions. Hence, this paper reports on the use of the gaseous plasma grafting method to load an acrylic acid (AA) group on the surface of a melt-blown non-woven PP fiber, which was further amidated to prepare a Cr(VI)-imprinted fiber. The parameters of the plasma grafting process were optimized, and the morphology, hydrophilicity, and surface structure of the fiber were analyzed. The adsorption performance and reuse efficiency of the ion-imprinted fiber toward the Cr(VI) anion are also discussed.

## 2. Experimental

### 2.1. Reagents and Instruments

A PP melt-blown non-woven fabric with a fiber diameter of 2–10 μm and specific surface area of 1.50 m^2^/g was homemade using a previously reported method [26]. Argon and air (purity of 99.99%) were supplied by Nanjing Shangyuan Industrial Gas Company (Nanjing, China). Other reagents, i.e., acrylic acid (AA), triethylenetetramine (TETA), HCl, acetone, ethanol, N, N-dimethylformamide (DMF), 1-hydroxybenzotriazole (HOBT), N, N’-dicycloethyl carbamide (DCC), formaldehyde, glutaral, epichlorohydrin, and sodium hydroxide were of analytic grade and used as received.

A pulsed radio frequency (RF) power source (500 W, MSY–I) and radio frequency matcher (2000 W, SP-II) were supplied by the Institute of Microelectronics, Chinese Academy of Sciences (Beijing, China). A rotary-vane vacuum pump (2X-8B, 1.1 kW) was supplied by Nanjing Vacuum Pump Co., Ltd (Nanjing, China).

### 2.2. Preparation of Ion-Imprinted Fibers

**Scheme 1:**
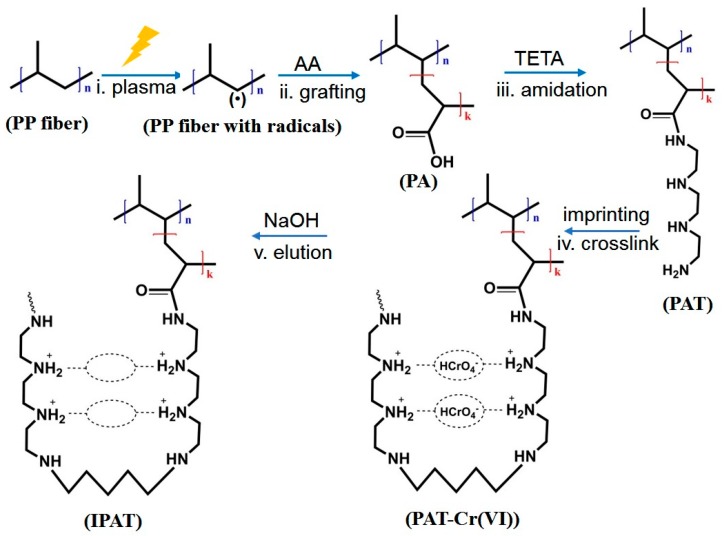
Schematic representation of the preparation process of ion-imprinted PP fibers (IPAT) (**i**: plasma treatment of PP fibers to form radicals; **ii**: grafting of acrylic acid (AA) on PP fibers to form AA-grafted PP (PA); **iii**: amidation of AA with triethylene tetramine (TETA) to form PAT; **iv**: imprinting of Cr(VI) template and crosslinking; **v**: elution of Cr(VI) template using a NaOH solution).

**Figure 1:**
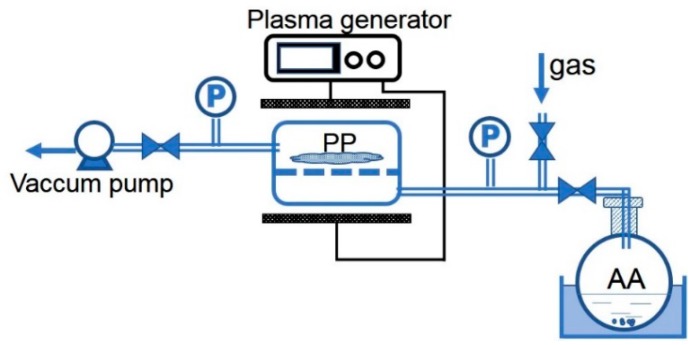
Illustration of the experiment setup of the plasma-induced polymerization of AA.

The reactions involved in the preparation of ion-imprinted fibers are shown in Scheme 1. First, AA was grafted onto the surface of a PP fiber [14,17]. As shown in Figure 1, three gases, namely AA, air, and argon, were used for the radio frequency discharge plasma irradiation to generate free radicals on PP fibers, respectively. Afterwards, AA gas was induced into the reactor at a certain pressure and temperature and grafted onto PP fibers through reaction with the free radicals. Then, the resultant AA-grafted PP fiber (PA) was amidated using 0.1 mol/L TETA in the medium of 50 mL DMF solution with DCC and HOBT concentrations of 0.1 and 0.15 mol/L, respectively. After grafting, the sample was washed three times with acetone and deionized water to remove the remaining reactants on the fiber surface. The amidated fiber (PAT) was obtained after drying in vacuum at 50 °C to a constant mass.

The PAT was weighed and soaked in 100 mL potassium dichromate solution (200 mg/L, pH = 3), followed by shaking and adsorption at 300 rpm for 2 h, and then immersed in a 50 mL aqueous solution of 30% TETA (pH = 3) for 30 min. The resulting fibers were crosslinked using 30% aqueous epoxy chloropropane, formaldehyde, and glutaraldehyde-ethanol solution at constant temperature, respectively. To remove template ions, the fibers were washed with 0.1 mol/L NaOH until Cr(VI) was not detected in the washing solution. After washing with deionized water and acetone, and drying at 50 °C to a constant mass, the Cr(VI)-imprinted fibers, denoted as IPAT, were obtained. At the same time, non-imprinted fibers (NIPAT) were prepared following the same preparation steps as for IPAT, except for the templating step, and used as a reference.

### 2.3. Analytic Methods

#### 2.3.1. Grafting Degree

The grafting degree of the PA fiber was determined by the titration method. The PA fiber was weighed and dissolved in 100 mL of xylene, then 20 mL of 0.05 mol/L NaOH-ethanol solution was added and refluxed for 30 min. After cooling to room temperature, titration with 0.05 mol/L HCl-isopropanol solution to the end point was performed using bromothymol blue as a pH indicator. The grafting degree, *Gr* (mmol/g), was calculated as:

*G_r_* = (*M*_1_ × *V*_1_ − *M*_2_ × *V*_2_)/*m* × 100% (1)

where *V*_1_ (mL) and *V*_2_ (mL) are the consumed volumes of NaOH and HCl solutions, respectively, *M*_1_ (mol/L) and *M*_2_ (mol/L) are the molar concentrations of NaOH and HCl solutions, respectively, and *m* (g) is the mass of PA. Each experiment was performed in triplicate. 

#### 2.3.2. Amino Content

The PAT was weighed and placed in a 100 mL conical flask with a stopper. An HCl-ethanol solution (50 mL) was added and shaken well. The flask was tightly sealed and immersed in a water bath at 40 °C for 2 h. After cooling to room temperature, 10 mL of solution was taken and placed in a new conical flask. After adding 10 mL of ethanol and 2 drops of phenolphthalein indicator solution, titration with the NaOH-ethanol solution to the end point was carried out. The amine group content, *E* (mmol/g), in the sample was calculated with Equation (2).

*E* = (50 × *C*_1_ − 5 × *C*_2_ × *V*_1_)/*m* (2)

where *C*_1_ (mol/L) is the concentration of the HCl-ethanol solution, *C*_2_ (mol/L) is the concentration of the NaOH–ethanol solution, *V*_1_ (mL) is the volume of the NaOH-ethanol solution consumed for the titration, and *m* (g) is the mass of PAT.

#### 2.3.3. Crosslink Degree of IPAT

The IPAT fiber sample was weighed, immersed in 50 mL acetic acid, and then soaked for 24 h. Then, it was washed three times with deionized water and dried to a constant mass at 50 °C. The crosslinking degree, *C* (%), was calculated as:

*C* = *m*_1_/*m*_0_ × 100% (3)

where *m*_1_ (g) is the mass of IPAT after soaking and drying, and *m*_0_ (g) is the mass of IPAT before soaking.

### 2.4. Characterizations

The fiber morphology was observed by scanning electron microscopy (SEM, S-3400N II, Hitachi, Japan). The surface functional groups of fibers were analyzed by Fourier transform infrared spectrometry (FTIR, NEXUS870, Nicolet, Japan). Elemental analyzer (Vario MACRO cube, Elementar, Germany) was used to evaluate the elemental composition (C, H, N, O) of the fibers. Non-woven fabric samples were cut into pieces of about 1 cm × 1 cm, pasted on the glass slide, and placed in the water contact angle tester (JC2000C, Shanghai Zhongchen Digital Technic Apparatus Co., Ltd, Shanghai, China). The water contact angle was measured by dripping about 1 L ultrapure water on the fiber surface.

### 2.5. Adsorption and Regeneration

The fibers were immersed into 100 mL of Cr(VI) solution (400 mg/L) in a shaker (300 rpm) and allowed to adsorb for 60 min. The Cr(VI) concentration before and after adsorption was determined on a inductively coupled plasma emission spectrometer (iCAP 6300, Thermo Fisher Scientific, Cambridge, UK). The adsorption capacity, *Q* (mg/g), was calculated as:

*Q* = 0.05 × (*C*_0_ − *C*_1_)/*m* (4)

where *C*_0_ (mg/L) and *C*_1_ (mg/L) are the concentrations of Cr(VI) before and after adsorption, respectively, and *m* (g) is the mass of fibers. 

The adsorption kinetics were fitted with pseudo-first-order (Equation (5)) and pseudo-second-order (Equation (6)) models.

(5) $$\ln(q_{e}-q_{t})=\ln q_{e}-k_{1}t$$

(6) $$\frac{t}{q_{t}}=\frac{1}{k_{2}q_{e}^{2}}+\frac{t}{q_{e}}$$

where *q_e_* and *q_t_* are the amount of adsorbed Cr(VI) at equilibrium and at an arbitrary time *t*, respectively, and *k_1_* (min^−1^) and *k_2_* (mg g^−1^ min^−1^) are the pseudo-first-order and pseudo-second-order rate constants of adsorption, respectively.

The adsorption isotherm was analyzed with the Langmuir and Freundlich models:

(7) $$\frac{C_{e}}{Q_{e}}=\frac{C_{e}}{Q_{m}}+\frac{1}{K_{L}Q_{m}}$$

(8) $$\ln Q_{e}=\ln K_{F}+\frac{1}{n}\ln C_{e}$$

where *C_e_* (mg/L) is the concentration of Cr(VI) in solution at equilibrium, *Q_e_* (mg/g) is the equilibrium adsorption capacity, *Q_m_* (mg/g) is the maximum adsorption capacity, *K*_L_ (L/mg) is the Langmuir constant, *K*_F_ is the Freundlich constant indicative of the adsorption capacity, and *n* is a parameter related to the sorption intensity.

After the adsorption of Cr(VI), the fibers were washed with 50 mL of NaOH solution (0.2%) for 30 min (150 rpm) to remove the adsorbed Cr(VI). The cleaning process was repeated until no Cr(VI) was detected in the washing solution. Elution efficiency, *D_n_* (%), was calculated as:

*D_n_* = *M_n_*/*Q_m_* × 100% (9)

where *n* is the number of washes, *M_n_* (mg) is the total amount of eluted Cr(VI) after *n* times elution, and *Q_m_* (mg) is the adsorption capacity of fibers. 

The regeneration efficiency, *R_n_,* of fibers was calculated as:

*R_n_* = *Q_n_*/*Q* × 100% (10)

where *Q*_n_ (mg/g) is the adsorption capacity of fibers after *n* cycles, and *Q* (mg/g) is the adsorption capacity of fibers in the first cycle.

To assess the adsorption selectivity of IPAT for Cr(VI), the fibers were placed into a mixed solution containing Cr(VI) (200 mg/L, pH = 3) and competing ions (phosphate, sulfate, and carbonate), and allowed to adsorb for 30 min. The adsorption selectivity, *S* (%), was calculated as:

*S* = *Q_n_*/*Q*_0_ × 100% (11)

where *Q_n_* (mg/g) and *Q*_0_ (mg/g) are the adsorption capacity of fibers towards Cr(VI) in the presence and absence of competing ions, respectively.

## 3. Results and Discussion

### 3.1. Optimization of Preparation Conditions

#### 3.1.1. Plasma Irradiation

**Figure 2:**
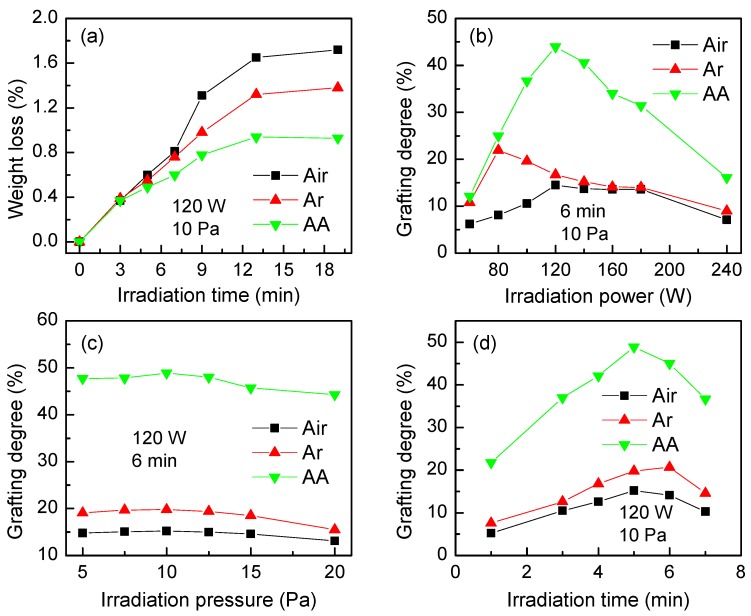
(**a**) The effect of the irradiation time on the weight loss of fibers and the effects of (**b**) the irradiation power, (**c**) pressure, and (**d**) time on the grafting degree of AA (grafting conditions: 150 Pa, 90 min, and 45 °C).

The etching effect of plasma irradiation on PP fibers and the effects of the applied power, plasma irradiation pressure, and irradiation time on the grafting degree of AA monomers are depicted in Figure 2. Figure 2a shows that the PP fibers lose weight under the three atmospheres (i.e., AA, Ar, and air) due to the plasma etching. After 13 min of irradiation, the high-energy particles in the plasma are consumed and the energy of particles in the atmosphere is reduced, so the etching effect on the PP fiber surface is weakened and no change in mass of fibers is noticed. The weight loss of PP fibers observed in the three gases at the same applied power (120 W) and pressure (10 Pa) follows the order of air > Ar > AA. This may be explained by the fact that the high-energy particles generated by air ionization have the maximum energy, while the particles generated by the AA monomer have the minimum energy. Particles with higher energy tend to bombard the material surface more violently and lead to higher mass loss. In general, the weight loss within 19 min was less than 2.0%, which could be ignored in the calculation of the grafting degree [17].

Figure 2b shows the effect of different powers on the AA grafting degree under the three gases. As can be seen, the grafting degree follows the order of AA > Ar > air. Unlike the latter two, when AA gas is used, active free radicals are generated by the ionization and fragmentation of the AA monomers, which are more likely to bind to the surface of the PP fibers and create active free radical sites. On the contrary, air and Ar atmospheres are more prone to etch the fiber surface, which is not favorable for the formation of free radicals. In addition, the oxygen in the air may remove the active free radical sites due to oxidation, which makes the grafting degree in the air atmosphere slightly lower than that in the inert Ar atmosphere [27]. As for grafting in an AA atmosphere, the applied power has an obvious effect. Hence, the grafting is favored until a power of 120 W, then the efficiency of grafting strongly decreases as the power increases from 120 to 240 W. The rationale for this behavior is related to the increase in dissociation degree of AA for a power between 70 and 120 W when the active particles move fast, increasing the number of free radicals on the surface of the PP fiber and favoring grafting of AA. When the discharge power exceeds 120 W, the etching effect generated by bombarding the PP surface is too large, so the grafting degree gradually decreases [28].

Figure 2c shows the effect of plasma irradiation pressure on the grafting degree efficiency. Compared with the applied power, the irradiation pressure has a slight influence on the grafting degree. Under an AA atmosphere, a grafting degree of almost 50% is seen between 5 and 12 Pa, which then decreases to 40% with the increase in irradiation pressure to 20 Pa. Figure 2d shows the effect of the irradiation time on the grafting degree of the PA fiber at 120 W and 10 Pa. Irrespective of the atmosphere used, an increase is noticed within the first 5–6 min of irradiation, but it then decreases. This behavior is explained by the saturation of the fiber surface with free radicals, which corresponds to the highest degree of grafting. When the discharge time is too long, the adjacent radicals are easily coupled and annihilated, a phenomenon associated with a decrease in grafting degree.

#### 3.1.2. Grafting Conditions

**Figure 3:**
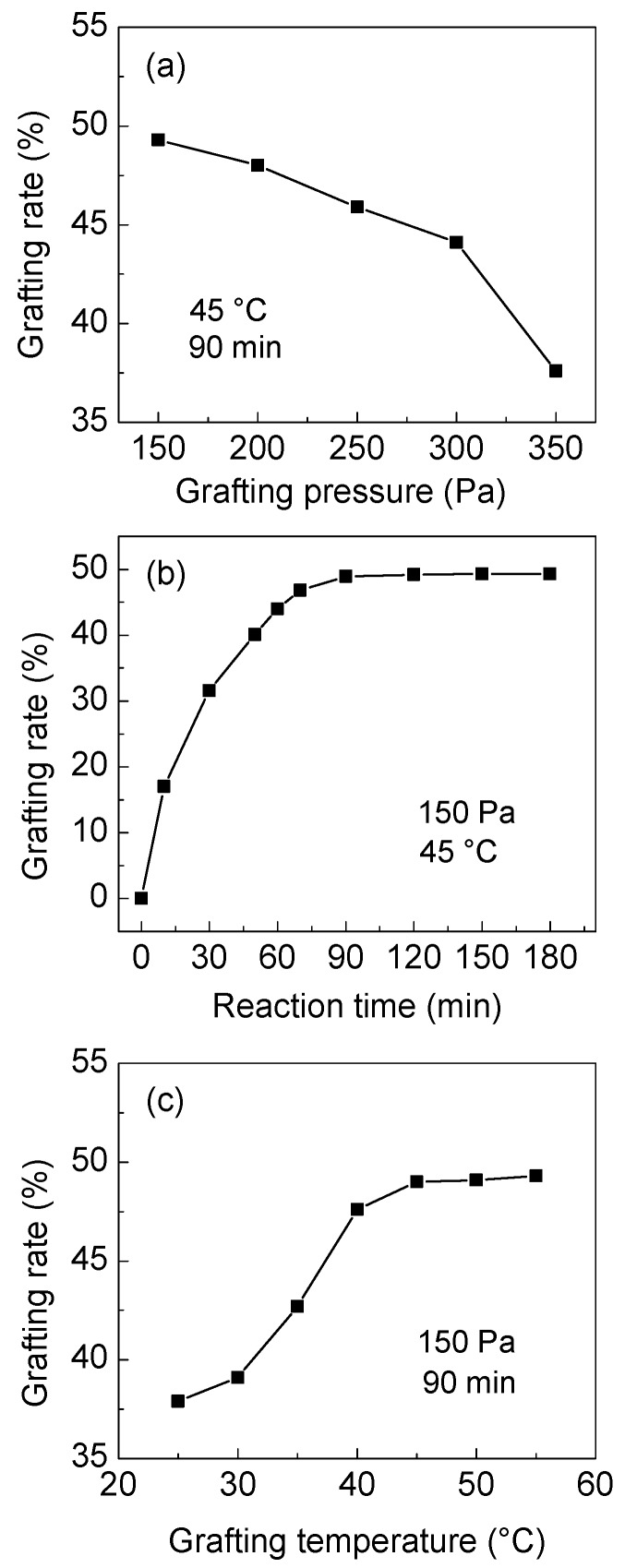
The effects of (**a**) the pressure, (**b**) reaction time, and (**c**) temperature on the grafting degree of PA (plasma irradiation conditions: 120 W, 10 Pa, and 5 min).

The effects of the gas pressure, duration and temperature on the grafting degree were studied, as shown in Figure 3a–c, respectively. As displayed in Figure 3a, the grafting degree of PA decreases with the increase in gas pressure. This is explained by the high concentration of AA in the reaction system when the pressure is low and thus, a higher number of monomers can be grafted on the surface active sites of PP fibers. The lowest pressure in this reaction system is controlled at 150 Pa using an AA atmosphere. The grafting degree of modified fibers increases with the extension of grafting time from 0 to 90 min, and then it is stable, as illustrated in Figure 3b. As the reaction progresses, the active sites on the surface of the fibers are consumed and the grafting reaction is gradually completed. As can be seen from Figure 3c, the reaction temperature increase results in an increase in grafting degree of PA in the temperature range of 25–45 °C, afterwards, it remains stable. In the first temperature range, when the temperature increases while the pressure is constant, the saturated vapor pressure of the AA monomer increases in the reaction system, as well as the concentration, enhancing the reaction rate and thus, the grafting degree. However, the grafting degree is stopped at temperatures higher than 45 °C because the number of active sites on the surface of the PP fiber is limited and the high temperature favors the self-polymerization of a fraction of the AA monomer.

#### 3.1.3. Amidation Efficiency of PAT and Grafting Degree of PA

**Figure 4:**
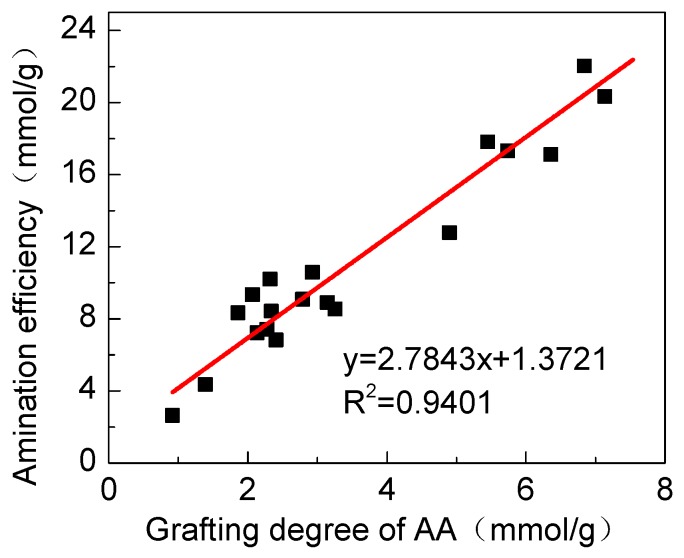
Relationship between the amidation efficiency of PAT and the grafting degree of PA.

Amidation of PA was carried out with different AA grafting degrees, and the relationship between the AA grafting degree of PA and the amidation efficiency of PAT is shown in Figure 4. From the first-order equation obtained by fitting, the concentration of the amino groups was 2.78 times higher than that of AA. According to the theoretical calculation, the highest number of amine groups on the polymer should be three times higher than that of AA. Hence, the amidation efficiency of the reaction is 92.7%.

#### 3.1.4. Crosslinking Reaction

**Figure 5:**
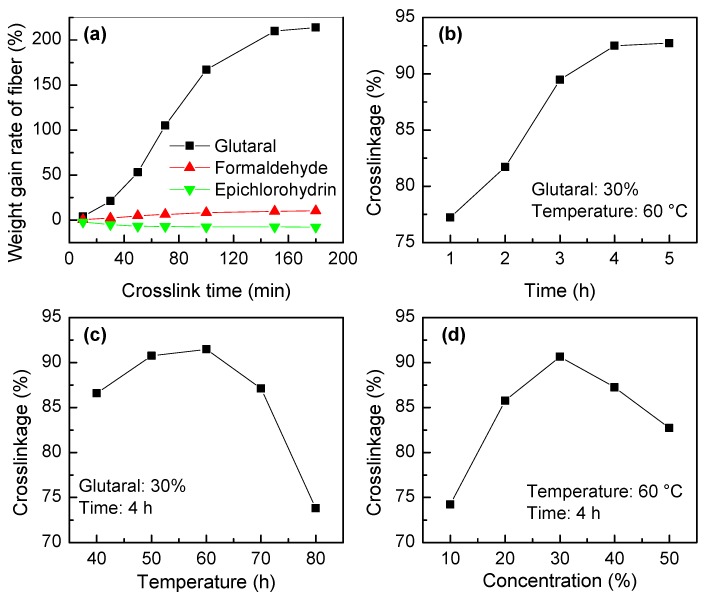
The effects of (**a**) the crosslinking agents, (**b**) time, (**c**) temperature, and (**d**) crosslinking agent concentration on the crosslinking degree of IPAT.

First, the effect of the crosslinking agent on the crosslinking degree was studied. Figure 5a shows the weight gain of fibers when formaldehyde, epichlorohydrin, and glutaraldehyde were used as crosslinking agents. When formaldehyde was used, the change in fiber weight was very small; only about 10% after 180 min of reaction. When epichlorohydrin was used as the crosslinking agent, the crosslinking effect was poor, and the fiber mass decreased by 7.8% after 180 min of crosslinking reaction. This effect is attributed to the ability of epichlorohydrin to desorb amines or AA chains on the surface of fibers. Among the three crosslinking agents, glutaraldehyde was the most appropriate, since an obvious gain of 214% was noticed after 180 min of reaction. According to the reaction scheme shown in Figure 1, two TETA molecules can be connected by glutaraldehyde during the cross-linking condensation reaction to bridge the polymeric spatial network.

Once the cross-linking agent was identified, i.e., glutaraldehyde, the factors affecting the crosslinking degree of IPAT were further investigated. According to Figure 5b, the increase in crosslinking time results in an increase in the crosslinking degree of IPAT, so that an optimum time of 4 h was established. As Figure 5c shows, increasing the temperature from 40 to 60 °C leads to an increase in the degree of crosslinking. At higher temperatures (60–80 °C), the cross-linking degree decreases significantly due to the partial loss of fiber structural organization. According to Figure 5d, the optimum amount of glutaraldehyde concentration is 30%. Therefore, the optimal crosslinking conditions for IPAT are 30% glutaraldehyde at 60 °C for 4 h of reaction.

### 3.2. Characterization

#### 3.2.1. SEM

**Figure 6:**
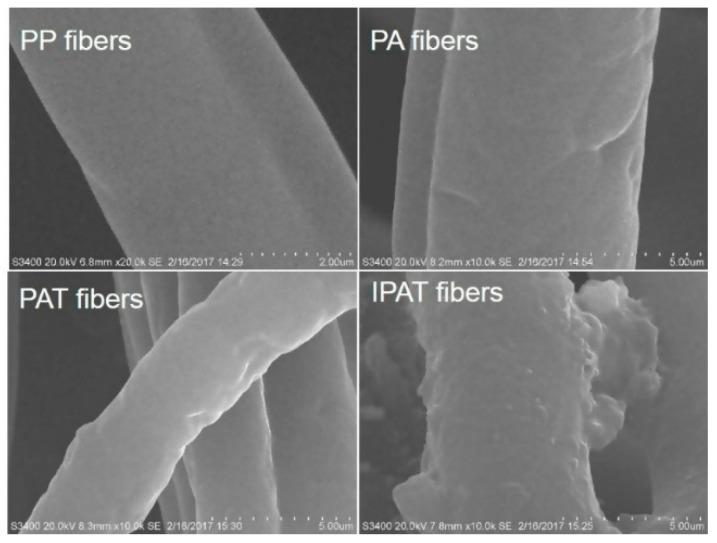
SEM images of PP, PA, PAT, and IPAT fibers.

Figure 6 shows SEM images of pristine and modified PP fibers. It can be seen from Figure 6 that the pristine PP fiber, whose diameter is in the micron range, displays a smooth surface. After gas phase grafting, the PA fiber surface is slightly rough, and corrugated layers can be seen. After amidation, the surface of PAT became rougher. After cross-linking with glutaraldehyde, the entire surface of the fiber is covered with a large number of granular polymers, thus denoting the formation of large local agglomerates. These results show that the fiber surface was successfully modified by grafting, amidation, and crosslinking. 

#### 3.2.2. FTIR

**Figure 7:**
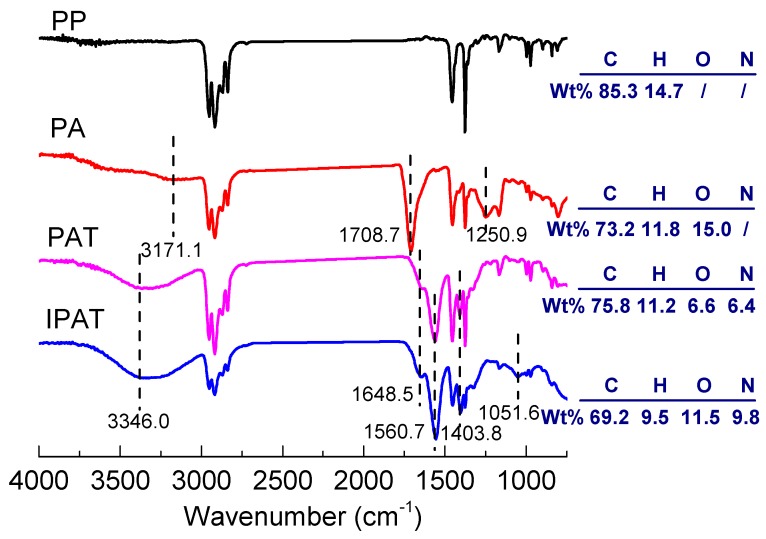
FTIR spectra and elemental analyses of PP, PA, PAT, and IPAT.

Figure 7 shows FTIR spectra of the fibers. The characteristic vibration bands of C–C, –CH_3_, and –CH_2_ could be observed only in the infrared spectrum of the pristine PP fiber. The vibration bands at 3171.1, 1708.7, and 1250.9 cm^−^^1^ displayed by the spectrum of PA are attributed to the stretching vibrations of –OH, C=O, and C–O, respectively, in the carboxyl group. After amidation, the band that appeared at 3346 cm^−^^1^ is the telescopic vibration of the –NH in the amide group. The bending vibration of –NH_2_ and the stretching vibration of C–N were identified at 1648.5 and 1403.8 cm^−^^1^, respectively. However, the strong vibration band at 1560.7 cm^−^^1^ is attributed to C=O in the amide group, which shifted to the right relative to the vibration band of C=O in the carboxyl group. This is because the π electrons in the amide group and the P electron conjugation on the nitrogen atom lead to the increase of C–N stretching vibration frequency. Compared with PAT, the intensity of the C–N telescopic vibration at 1051.6 cm^−^^1^ (mostly in the fingerprint area) increased in the spectrum of IPAT. On the other hand, the elemental analysis showed a large amount of the N element in the PAT fibers, which indicates successful amidation of the fiber surface. The content of the N element in IPAT increased significantly, pointing out that a larger amount of TETA was fixed on the surface of imprinted fibers by the crosslinking reaction. In addition, the content of the O element significantly increased as a result of the hydroxyl groups generated on the fiber by the acetal reaction between glutaraldehyde and TETA.

#### 3.2.3. Water Contact Angle

**Figure 8:**
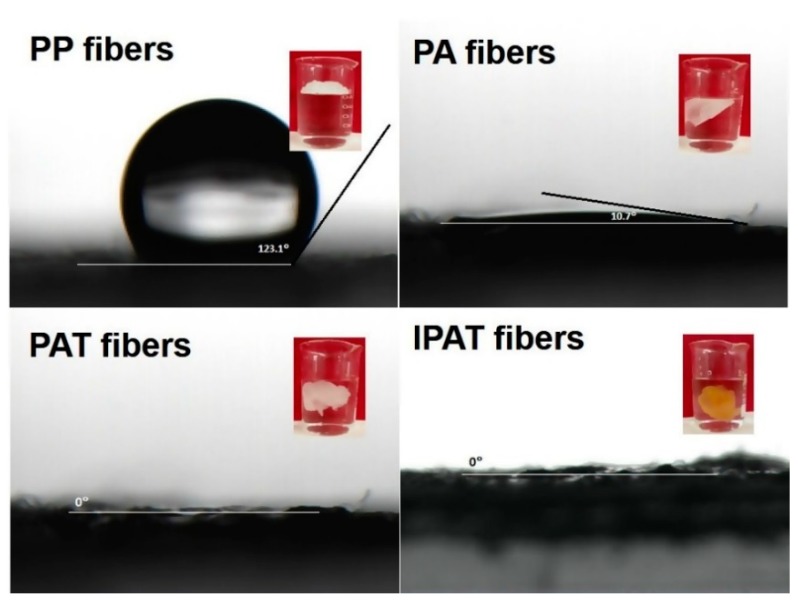
Water contact angle and hydrophilicity analysis of PP, PA, PAT, and IPAT.

The change in water contact angle of fibers is depicted in Figure 8. Compared with PP fibers, the water contact angle of PA fibers decreased significantly from 123.1° to 10.7°, indicating that grafting with the AA monomer significantly improved the hydrophilicity of the PP fibers. It can be seen from the illustration inset that PP fibers float on the water surface due to hydrophobicity, while PA fibers can be immersed in water after grafting with the AA monomer. The hydrophilicity of PAT and IPAT was further increased, and the water contact angle was reduced from 10.7° to nearly 0°, i.e., water droplets were immediately adsorbed on the fabric surface. The amines grafted on the surface of the fiber through the amidation reaction enhanced the surface polarity of the fibers, resulting in an enhanced hydrophilicity, which is conducive to the adsorption process in an aqueous solution.

### 3.3. Adsorption of Cr(VI)

#### 3.3.1. Effect of pH on the Adsorption Capacity of IPAT

**Figure 9:**
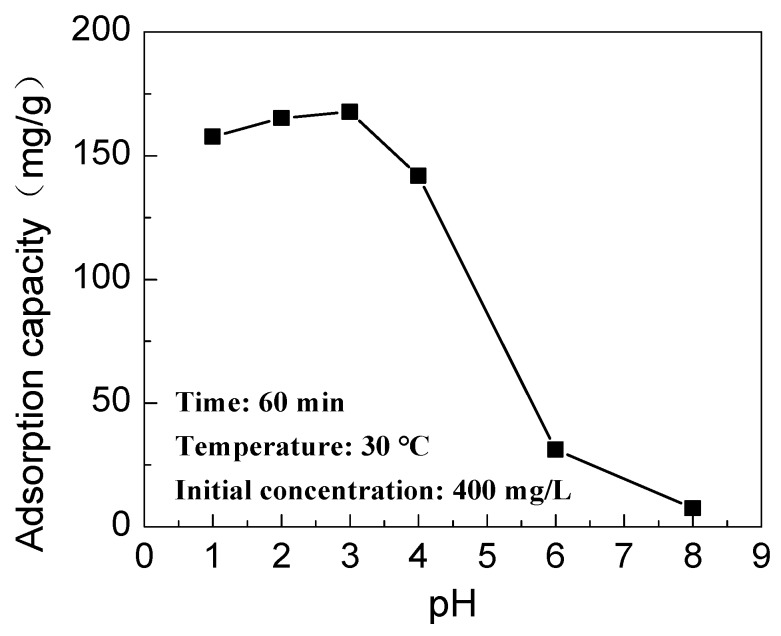
The effect of the pH on the adsorption capacity of IPAT towards Cr(VI).

The effect of the pH on the adsorption of Cr(VI) on IPAT is shown in Figure 9. In the pH range of 1–3, the adsorption capacity of IPAT to Cr(VI) is high with a slight increase tendency, reaching the maximum adsorption capacity of about 167 mg/g at pH = 3. As the pH continues to increase, the adsorption capacity of the fiber decreases rapidly, and when the pH value is 8, the adsorption capacity drops to about 10 mg/g. When the solution is acidic, the protons in solution easily combine with the lone pair electrons of the N in the amino groups on the surface of IPAT, making the amine groups positively charged. As Cr(VI) is an anion in solution, the adsorption on the protonated amine groups is facilitated by electrostatic forces [29]. Meanwhile, IPAT was prepared at pH = 3, so the microstructure of imprinted areas is favorable for the adsorption of Cr(VI). When the pH is less than 3, part of HCrO_4_^−^ is converted into non-ionic H_2_CrO_4_, which is difficult to bind to the protonated amine groups, so the adsorption capacity of the fiber is slightly reduced. When the pH is higher than 3 and increases gradually, the amine groups on the fiber surface are deprotonated and negatively charged, which negatively impacts the adsorption of Cr(VI).

#### 3.3.2. Adsorption Kinetics

**Figure 10:**
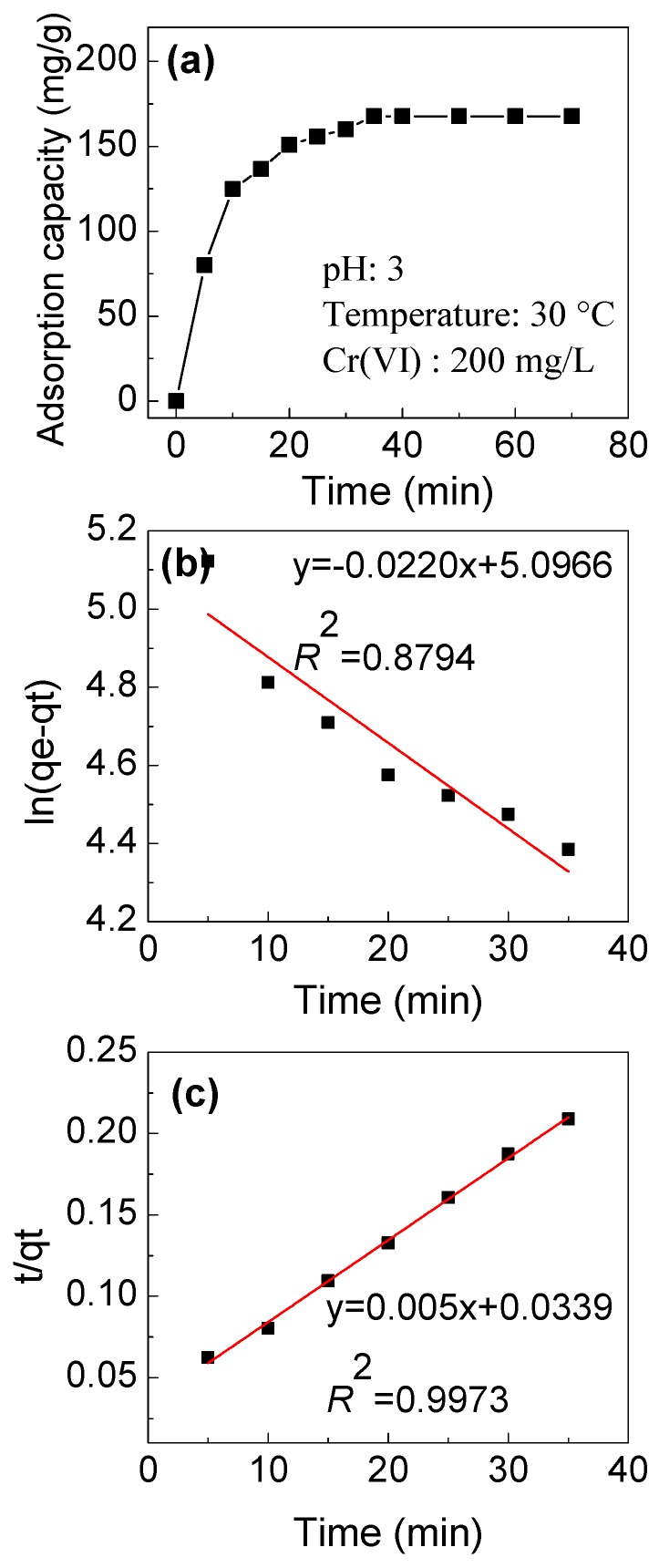
(**a**) The kinetics curve of Cr(VI) adsorption onto IPAT and the linearized (**b**) pseudo-first-order and (**c**) pseudo-second-order kinetics.

The kinetics for the adsorption of Cr(VI) onto IPAT was studied and the results are illustrated in Figure 10a. The adsorption of Cr(VI) was very fast in the first 10 min of adsorption. As the functional groups of fiber materials are evenly distributed on the surface and all available for the adsorption, the saturation is achieved in a short time. After 35min, the surface amine groups are all occupied by Cr(VI), and the sorption process reaches equilibrium. The linear fitting results and parameters are shown in Figure 10b,c. Generally, the adsorption process that conforms to the pseudo-first-order kinetic model is considered to be physical adsorption, and the adsorption rate is proportional to the number of adsorption sites on the surface of the adsorbent. The adsorption process of the pseudo-second-order kinetic model is a chemical adsorption process. The surface of the adsorbent and Cr(VI) achieve a chemical adsorption by sharing electron pairs, charge exchange, or electrostatic attraction. The *R*^2^ of the pseudo-first-order kinetic model is 0.8794, while for the pseudo-second-order kinetic model, *R*^2^ is 0.9973. Hence, the adsorption of Cr(VI) on fibers is better fitted by the pseudo-second-order kinetic model, indicating that the adsorption of Cr(VI) is controlled by chemical phenomena [30]. Hence, the electrostatic attraction between the protonated amine groups and Cr(VI) anions under acidic conditions is the main driving force for Cr(VI) adsorption on the modified fibers.

#### 3.3.3. Adsorption Isotherm

**Figure 11:**
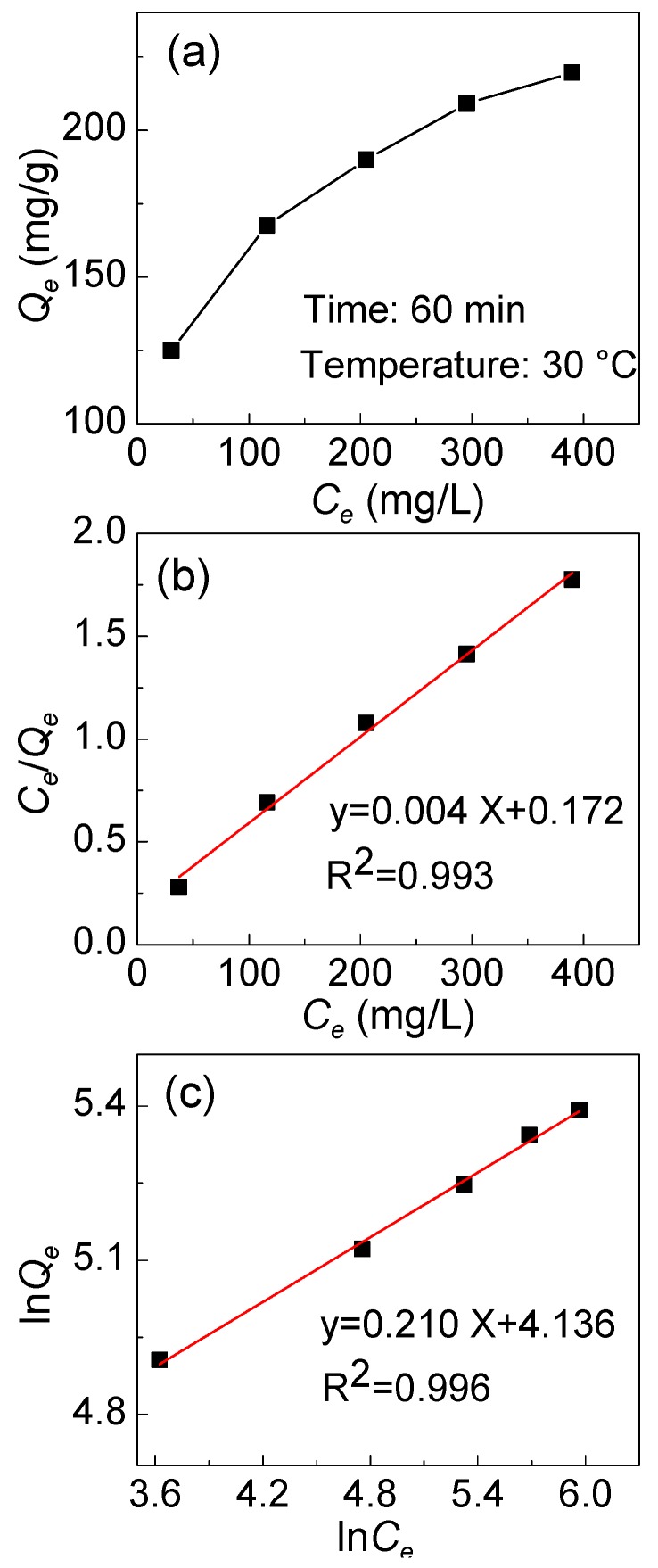
(**a**) The effect of the initial Cr(VI) concentration on the adsorption capacity of Cr(VI) onto IPAT and the linearization of the (**b**) Langmuir and (**c**) Freundlich models.

The adsorption isotherm was drawn based on the results obtained using different initial concentrations of Cr(VI) (30–400mg/L). As can be seen from Figure 11a, the adsorption capacity of IPAT increased with the increase in initial Cr(VI) concentration. The linearized versions of the two models are illustrated in Figure 11b,c and the values of the kinetic parameters are listed in Table 1. It was found that the *R*^2^ value of Langmuir and Freundlich was 0.993 and 0.996, respectively. Although both models fit the data, the adsorption of Cr(VI) is better fitted by the Freundlich model, showing a multilayer adsorption, and the adsorption process varies at different areas on the surface of the fiber. In the Freundlich model, the value of *n* is 4.76, indicative of an favorable adsorption of Cr(VI) onto the surface of IPAT fibers [31].

Comparisons between maximum adsorption capacities (*q*_max_) of IPAT and other ion imprinted adsorbents for Cr(VI) reported in the literature are presented in Table 2. The results show that IPAT exhibits a reasonable capacity for Cr(VI) adsorption from aqueous solutions.

#### 3.3.4. Adsorption Selectivity

**Figure 12:**
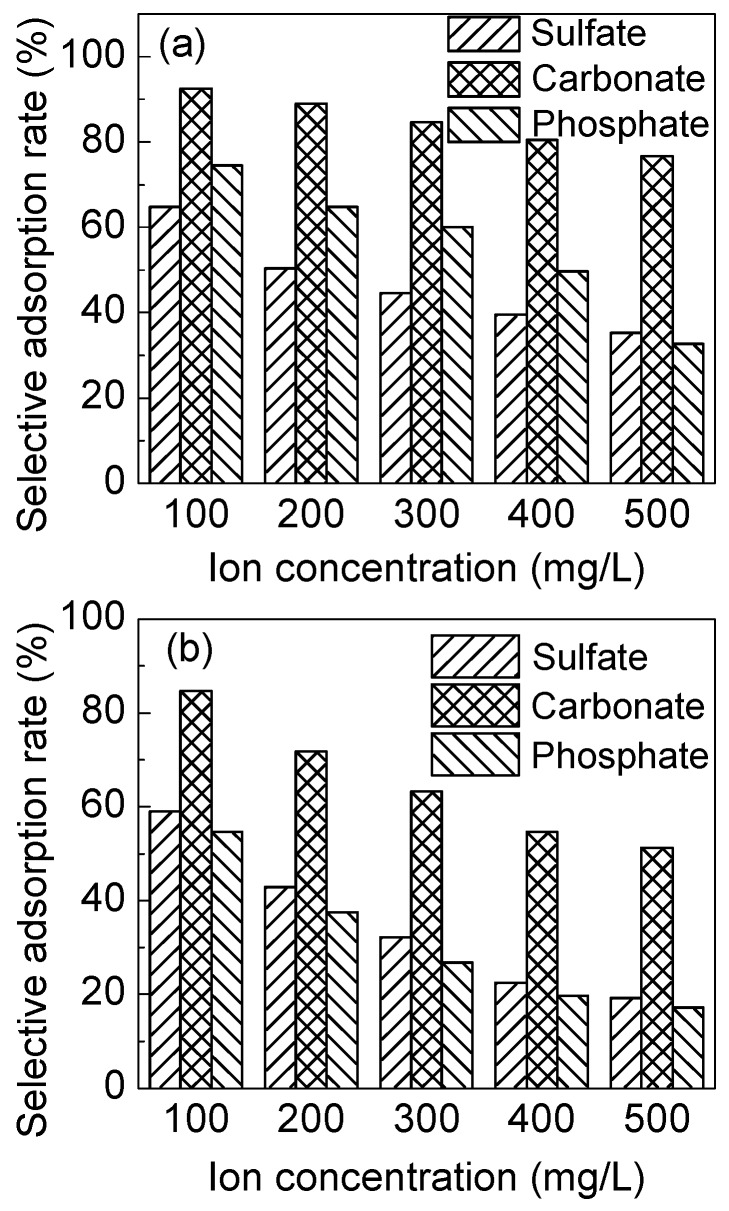
The effects of the competing ions on the adsorption selectivity of (**a**) IPAT and (**b**) NIPAT.

It can be seen from Figure 12a that the adsorption capacity of IPAT for Cr(VI) decreases as the concentration of competing ions (i.e., phosphate, sulfate, and carbonate) increases, which demonstrates that the competing ions reduce the adsorption capacity of fibers for Cr(VI) due to the similar affinity for the adsorption sites on the fiber surface. The strength of adsorption of the three competing ions increases in the order of phosphate > sulfate > carbonate, among which phosphate and sulfate have a greater effect on the adsorption of Cr(VI) than that of carbonate. When the concentration of the three competing ions (i.e., phosphate, sulfate, and carbonate) is 2.5 times higher than that of Cr(VI), the adsorption selectivity of IPAT fibers towards Cr(VI) is 32.7, 35.3, and 76.7%, respectively. Compared with IPAT, the adsorption selectivity of NIPAT is significantly lower. When the concentration of three ions (i.e., phosphate, sulfate, and carbonate) is 2.5 times higher than that of Cr(VI), the adsorption selectivity of NIPAT fibers to Cr(VI) is 17.3, 19.3, and 51.2%, respectively.

#### 3.3.5. Desorption and Regeneration

**Figure 13:**
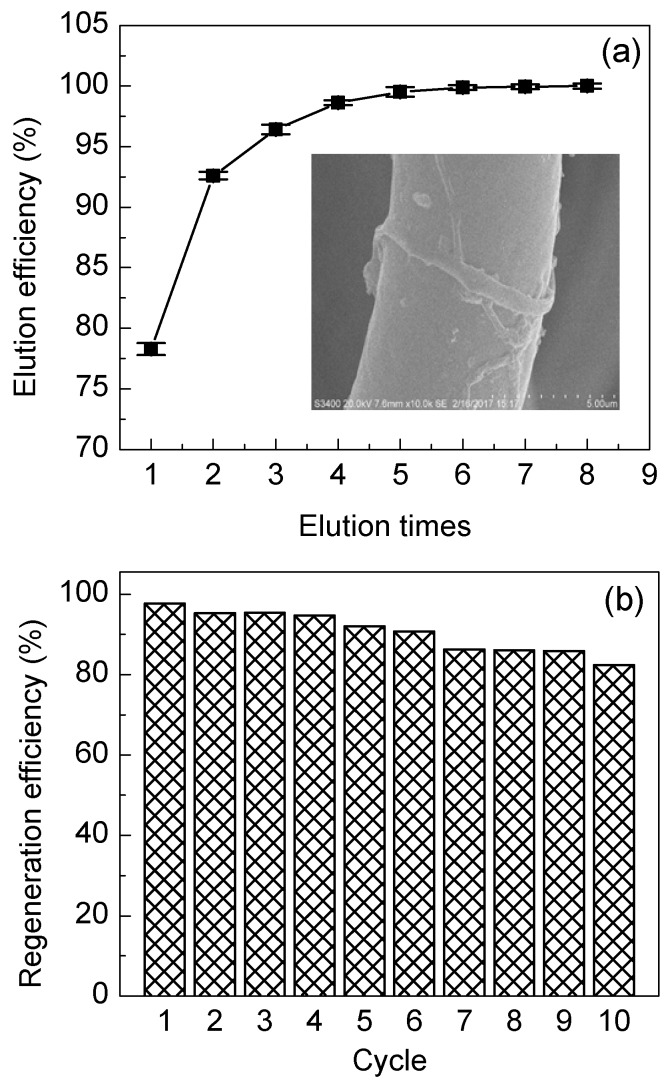
(**a**) The elution efficiency after several elution cycles and (**b**) the regeneration efficiency of IPAT after 10 adsorption–desorption cycles.

It can be seen from Figure 13a that nearly 80% of Cr(VI) can be removed after the first desorption cycle, the adsorbed Cr(VI) being almost completely removed after five cycles, indicating that NaOH solution has a good elution effect on the adsorbed Cr(VI) on the fiber. Under acidic conditions, the positively charged amine groups on the surface of the fibers attract the negatively charged Cr(VI) anions thus promoting the adsorption process. On the other hand, the OH^−^ in sodium hydroxide solution has a stronger affinity to protons compared with the amine group and can quickly capture the H^+^ in the protonated amine groups. As a result, the amine groups loss the ability to bind the Cr(VI) anion and achieve the purpose of regeneration. It can be seen from the SEM images taken for the fibers after elution (insert in Figure 13a) that the fiber surface displays the morphology of the fibers before adsorption, which demonstrates that the adsorption and elution process has no effect on the morphostructural properties of the fibers. Figure 13b shows that the adsorption efficiency of fibers stays above 82% after 10 adsorption and regeneration cycles. This kind of adsorption strength is moderate, neither too small to decrease adsorption quantity, nor too strong to make the elution difficult.

#### 3.3.6. The Effect of the Oxidation of Cr(VI) on the IPAT Structural Integrity

**Figure 14:**
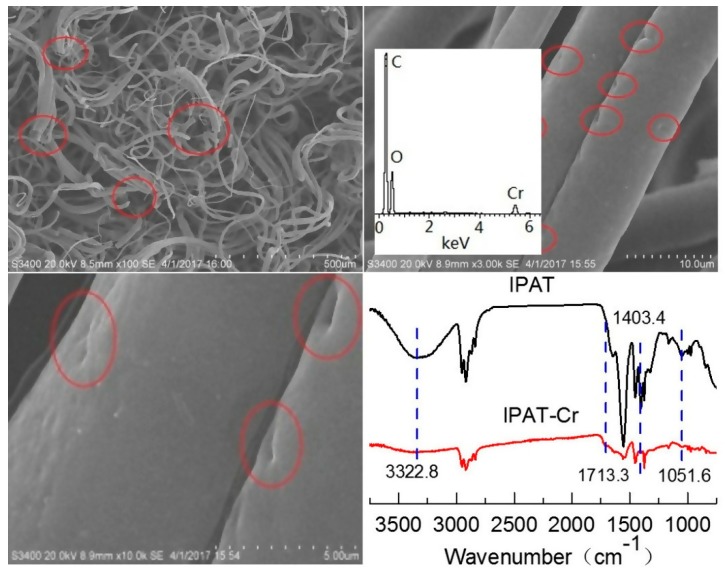
SEM images and FTIR spectra of dried IPAT loaded with Cr(VI).

After adsorption of Cr(VI) and drying to a constant weight, the morphostructural properties of IPAT were analyzed by SEM and FTIR spectroscopy. As SEM images displayed in Figure 14 show, a large number of fibers were fractured, and deep holes appeared on their surface, which can explain the fiber fracture. In addition, the color of the fibers changed from yellow to dark brown, while the fibers were brittle with almost no hardiness. A large amount of Cr(VI) was found on the surface of the fiber via EDS analysis. The FTIR spectra of IPAT fibers before and after adsorption of Cr(VI) show that the characteristic bands of the original PP fiber remain basically unchanged. However, the bands assigned to an amide group (3322.8, 1403.4, and 1051.6 cm^−1^) almost disappeared, whereas the vibration band of –C=O at 1556 cm^−1^ was significantly weakened, which reveals the damage to the surface functional amide groups. Meanwhile, the corresponding absorption band for the existing Cr(VI) was found at 1713.4 cm^−1^ in the FTIR spectrum [38]. Therefore, after adsorption of Cr(VI) and drying, the structural integrity of the IPAT fibers formed by amidation and crosslinking was destroyed due to the strong oxidizing effect of Cr(VI). Compared with the morphology of IPAT in Figure 13a, it can be concluded that the oxidation and damage of fibers caused by Cr(VI) only occurred when they were subjected to drying without elution, whereas they exhibited resistance to Cr(VI) oxidation under normal conditions of use, i.e., adsorption, elution, and the regeneration procedure.

## 4. Conclusions

In this work, a non-thermal RF plasma treatment followed by gaseous phase AA grafting was used to prepare ion-imprinted PP fibers. The grafting degree was the highest when AA was used as a plasma atmosphere, and the optimum treatment conditions were 5 min, 120 W and 10 Pa. The output power and processing time had a greater impact on the grafting degree than the monomer pressure. The highest grafting degree of AA was reached under the condition of 150 Pa, 90 min, and 45 °C. When glutaraldehyde (30%) was used as a crosslinking agent, the highest crosslinking degree was obtained at 60 °C for 4 h. SEM and FTIR results indicated that the imprinted fibers were successfully prepared by a step-by-step method. Meanwhile, the hydrophilicity of imprinted fibers was greatly enhanced compared with that of PP fibers, which is conducive to the adsorption process in aqueous solution. The adsorption performance of the obtained IPAT fibers was evaluated in Cr(VI) removal. The highest adsorption capacity of 167 mg/g was obtained at pH = 3. The kinetics of adsorption followed the pseudo-second-order model and the adsorption isotherm data were better fitted by the Freundlich model. The adsorption selectivity of IPAT and NIPAT was significantly affected by phosphate and sulfate, while the carbonate ion had a slight effect. In addition, in the presence of competing ions, IPAT showed higher adsorption selectivity compared with NIPAT. The adsorbed Cr(VI) was eluted rapidly and effectively by a NaOH solution (0.2%). After 10 regenerative adsorption experiments, the adsorption efficiency of IPAT was still higher than 80%, confirming the reusability of the prepared fibers. Under normal conditions of use, IPAT exhibits efficient resistance to Cr(VI) oxidation, which makes it feasible for use in adsorption.

## Figures and Tables

**Table 1:** Langmuir and Freundlich isotherm parameters for Cr(VI) adsorption onto IPAT.

Langmuir			Freundlich		
*Q_m_* (mg/g)	*K*_L_ (L/mg)	*R* ^2^	*n*	*K*_F_ (mg/g)	*R* ^2^
238.1	0.024	0.993	4.76	62.8	0.996

**Table 2:** Adsorption capacities of various ion imprinted adsorbents for Cr(VI).

Adsorbents	Morphology	*q_max_* (mg/g)	References
styrene/4-VP/EDGMA	Particles 53–90 μm	37.58	[32]
4-VP/HEMA	Particles 75–150 μm	172.12	[33]
Magnetic poly(4-VP)	-	6.20	[34]
4-VP/EDGMA	Particles 50–100 μm	286.56	[35]
SCC/Epichlorohydrin	-	177.62	[36]
Magnetic	Particles ~70 nm	39.3	[29]
Pebax/chitosan/GO/APTES	Nanofibers ~90 nm	204 5	[37]
IPAT	Fiberous ~7 μm	167	This work

4-VP: 4-vinylpyridine; EGDMA: ethylene glycol dimethacrylate; HEMA: hydroxyethyl methacrylate; HEMAH: 2-methacryloylamido histidine; SCC: sodium carboxymethyl cellulose; GO: graphene oxide; APTES: 3-aminopropyltriethoxysilane.

## References

[1] Kimbrough D.E., Cohen Y. (1999). A critical assessment of chromium in the environment. Crit. Rev. Environ. Control.

[2] Dubey S.P., Gopal K. (2007). Adsorption of chromium(VI) on low cost adsorbents derived from agricultural waste material: A comparative study. J. Hazard. Mater..

[3] Erdem M., Tumen F. (2004). Chromium removal from aqueous solution by the ferrite process. J. Hazard. Mater..

[4] Ho W.S.W., Poddar T.K. (2000). New membrane technology for removal and recovery of chromium from waste waters. Environ. Prog. Sustain..

[5] Luo S., Qin F. (2017). Fabrication uniform hollow Bi_2_S_3_ nanospheres via Kirkendall effect for photocatalytic reduction of Cr(VI) in electroplating industry wastewater. J. Hazard. Mater..

[6] Mohanty K., Jha M. (2006). Biosorption of Cr(VI) from aqueous solutions by *Eichhornia crassipes*. Chem. Eng. J..

[7] Wang W., Li M. (2012). Column adsorption of chromium(VI) by strong alkaline anion-exchange fiber. J. Appl. Polym. Sci..

[8] Dognani G., Hadi P. (2019). Effective chromium removal from water by polyaniline-coated electrospun adsorbent membrane. Chem. Eng. J..

[9] Hayashi N., Chen J. (2018). Nitrogen-containing fabric adsorbents prepared by radiation grafting for removal of chromium from wastewater. Polymers.

[10] Huang R., Ma X. (2018). A novel ion-imprinted polymer based on graphene oxide-mesoporous silica nanosheet for fast and efficient removal of chromium (VI) from aqueous solution. J. Colloid Interface Sci..

[11] Wei Q.F., Mather R.R. (2005). Functional nanostructures generated by plasma-enhanced modification of polypropylene fibre surfaces. J. Mater. Sci..

[12] Gul Dincmen M., Hauser P.J. (2016). Plasma induced graft polymerization of three hydrophilic monomers on nylon 6,6 Fabrics for enhancing antistatic property. Plasma Chem. Plasma Proc..

[13] Basarir F., Choi E.Y. (2005). Electrochemical properties of PP membranes with plasma polymer coatings of acrylic acid. J. Membrane Sci..

[14] Chen H., Guo M. (2018). Green and efficient synthesis of an adsorbent fiber by plasma-induced grafting of glycidyl methacrylate and its Cd(II) adsorption performance. Fibers Polym..

[15] Tseng C., Wang C. (2006). Polypropylene fibers modified by plasma treatment for preparation of Ag nanoparticles. J. Phys. Chem. B.

[16] Haji A., Shoushtari A.M. (2019). Grafting of poly(propylene imine) dendrimer on polypropylene nonwoven: Preparation optimization, characterization, and application. Fibers Polym..

[17] Luo Z., Chen H. (2018). Surface grafting of styrene on polypropylene by argon plasma and its adsorption and regeneration of BTX. J. Appl. Polym. Sci..

[18] Noor S., Xiakeer S. (2018). Controlled levofloxacin release and antibacterial properties of β-cyclodextrins-grafted polypropylene mesh devices for hernia repair. Polymers.

[19] Khlyustova A., Galmiz O. (2015). Underwater discharge plasma-induced coating of poly(acrylic acid) on polypropylene fiber. J. Mater. Sci..

[20] Buček A., Popelka A. (2016). Acrylic acid plasma treatment of polypropylene nonwoven fabric. Fibres Text. East. Eur..

[21] Sciarratta V., Vohrer U. (2003). Plasma functionalization of polypropylene with acrylic acid. Surf. Coat. Technol..

[22] Nishide H., Tsuchida E. (1976). Selective adsorption of metal ions on poly(4-vinylpyridine) resins in which the ligand chain is immobilized by crosslinking. Macromol. Chem. Phys..

[23] Li T., Chen S. (2011). Preparation of an ion-imprinted fiber for the selective removal of Cu^2+^. Langmuir.

[24] Zhang L., Yang S. (2017). Surface ion-imprinted polypropylene nonwoven fabric for potential uranium seawater extraction with high selectivity over vanadium. Ind. Eng. Chem. Res..

[25] Yang S., Ji G. (2019). Polypropylene nonwoven fabric modified with oxime and guanidine for antibiofouling and highly selective uranium recovery from seawater. J. Radioanal. Nucl. Chem..

[26] Guo M., Liang H. (2016). Study on melt-blown processing, web structure of polypropylene nonwovens and its BTX adsorption. Fibers Polym..

[27] Černáková L., Kováčik D. (2005). Surface modification of polypropylene non-woven fabrics by atmospheric-pressure plasma activation followed by acrylic acid grafting. Plasma Chem. Plasma Proc..

[28] Altay L., Bozaci E. (2019). The effect of atmospheric plasma treatment of recycled carbon fiber at different plasma powers on recycled carbon fiber and its polypropylene composites. J. Appl. Polym. Sci..

[29] Hassanpour S., Taghizadeh M. (2018). Magnetic Cr(VI) ion imprinted polymer for the fast selective adsorption of Cr(VI) from aqueous solution. J. Polym. Environ..

[30] Wu S., Dai X. (2018). Highly sensitive and selective ion-imprinted polymers based on one-step electrodeposition of chitosan-graphene nanocomposites for the determination of Cr(VI). Carbohydr. Polym..

[31] Xia L., Huang Z. (2018). Bagasse cellulose grafted with an amino-terminated hyperbranched polymer for the removal of Cr(VI) from aqueous solution. Polymers.

[32] Pakade V., Cukrowska E. (2011). Selective removal of chromium (VI) from sulphates and other metal anions using an ion-imprinted polymer. Water Sa.

[33] Bayramoglu G., Arica M.Y. (2011). Synthesis of Cr(VI)-imprinted poly(4-vinyl pyridine-co-hydroxyethyl methacrylate) particles: Its adsorption propensity to Cr(VI). J. Hazard. Mater..

[34] Tavengwa N.T., Cukrowska E. (2013). Synthesis, adsorption and selectivity studies of N-propyl quaternized magnetic poly(4-vinylpyridine) for hexavalent chromium. Talanta.

[35] Kong D., Zhang F. (2014). Fast removal of Cr(VI) from aqueous solution using Cr(VI)-imprinted polymer particles. Ind. Eng. Chem. Res..

[36] Velempini T., Pillay K. (2017). Epichlorohydrin crosslinked carboxymethyl cellulose-ethylenediamine imprinted polymer for the selective uptake of Cr(VI). Int. J. Biol. Macromol..

[37] Etemadi M., Samadi S. (2017). Selective adsorption of Cr(VI) ions from aqueous solutions using Cr^6+^-imprinted Pebax/chitosan/GO/APTES nanofibrous adsorbent. Int. J. Biol. Macromol..

[38] Ren Z., Xu X. (2016). FTIR, Raman, and XPS analysis during phosphate, nitrate and Cr(VI) removal by amine cross-linking biosorbent. J. Colloid Interf. Sci..

